# Basic Characteristics of Oligodendrogliomas at the Shohada-e Tajrish Hospital (2008 to 2014)

**Published:** 2017-07-01

**Authors:** Mahsa Ahadi, Afshin Moradi, Azadeh Rakhshan, Alireza Arefian, Mitra Rafizadeh, Hanieh Zham

**Affiliations:** 1 *Cancer Research Center, Shohada-e Tajrish Hospital, Shahid Beheshti University of Medical Sciences, Tehran, Iran*

**Keywords:** Oligodendrogliomas, Central nervous system, Location, Grade, Age

## Abstract

**Background and Objectives:**

Gliomas are the most prevalent subgroup of primary brain tumors with a relatively high mortality. However, oligodendrogliomas have a better prognosis compared to other subtypes due to their sensitivity to chemotherapy. Considering the low incidence and the resulting lack of information about oligodendrogliomas, particularly in Iran, this study aimed at assessing their basic characteristics.

**Methods:**

In this descriptive retrospective study, patients with definite diagnosis of oligodendroglioma were identified by reviewing the archives of pathology reports at the department of pathology of Shohada-e Tajrish Hospital during years 2008 to 2014. Age, gender, location, and the grade of the tumor were extracted and entered to the SPSS statistical software for analysis.

**Results:**

A total of 182 patients, including 115 males (63.2%) and 67 females (36.8%), were included with a mean age of 38.5±13.36 years. Frontal lobe was involved in 53 patients (29.1%), parietal lobe in 31 (17.0%), temporal lobe in 22 (12.1%), frontoparietal area in 15 (8.2%), parieto-occipital area in 11 (6.0%), temporoparietal and frontotemporal areas each in 9 subjects (4.9%), occipital lobe in 5 (2.7%), and the brainstem in 4 (2.2%). Furthermore, 108 cases (59.3%) had grade-2 and the remaining74 patients (40.7%) had grade-3 anaplastic oligodendrogliomas. The mean age of subjects with brainstem oligodendrogliomas was significantly lower than the other patients (p=0.025).

**Conclusion:**

Oligodendrogliomas commonly effects the frontal lobe, followed by the parietal and temporal lobes. The mean age of subjects with brainstem lesions was significantly lower than other patients. Age, gender or location of the tumor did not independently predict a higher grade lesion.

## Introduction

Gliomas are the most prevalent subgroup of primary brain tumors, a heterogeneous group of uncommon brain cancers with a relatively high mortality. However, there is a rare type among these tumors that are considered to have a better prognosis compared to other subtypes, due to their sensitivity to chemotherapy, known as Oligodendrogliomas (1-10). Recently, a correlation has been found between the response of these tumors to chemotherapy with certain genetic anomalies, including loss of 1p and 19q (1, 5, 7, 8, 11).

Oligodendrogliomas commonly effect adults aged 40 to 60 years old, and a slight male preponderance has been observed in these cases (12-14). The slowly progressing tumors usually originate from the white matter of the brain and the frontal lobe is involved in more than half of the cases. The characteristic diffusely infiltrative growth of oligodendrogliomas leads to a high risk of seizures in patients during the course of their disease (12, 15-17). No risk factors have been identified for these tumors, yet, a few cases have been found with a history of radiation for other reasons (18).

The incidence rate reported for oligodendro-gliomas of all gliomas ranges from 4% to 33% in different studies (4, 18-22). The differences between previous reports could be attributed to improvements in diagnosis of tumors due to an increased accessibility to diagnostic scans and greater knowledge about the prognosis of the disease. Changes in definition, classification and diagnosis of these tumors might be another reason for the wide range in the documented incidence rates (20, 22). 

Considering the low incidence of oligodend-rogliomas and the resulting lack of information on this subject, particularly in Iran, the present study aimed at providing basic characteristics of oligodendroglioma tumors diagnosed in patients referred to Shohada-e Tajrish Hospital during a 7-year period from 2008 to 2014. 

## Methods

In this descriptive retrospective study, patients with definite diagnosis of oligodendroglioma, determined by histopathological evaluations, were identified by reviewing the archives of pathology reports documented at the department of pathology of Shohada-e Tajrish Hospital during years 2008 to 2014. Age, gender, location and grade of the tumor were extracted from medical records and entered in the SPSS software v.22 (23) for statistical analysis. The extracted data were used anonymously and the patients’ information was regarded confidential throughout the research. The study protocol was evaluated and approved by the Ethics Committee of Shahid Beheshti University of Medical Sciences. Descriptive results were presented as frequencies and percentages for gender, location and grade of the tumor, and as mean and standard deviation for age. The relationship between qualitative variables was evaluated by Chi-square test and Fisher’s exact test if required. Independent samples T test was used to compare the difference in age of patients based on their gender and grade of their tumor. Analysis of Variance (ANOVA) test with Tukey’s HSD post hoc test were also used to compare the mean age of patients based on the location of their tumor. Finally, binary logistic regression analysis was applied to identify the independent risk factors for high-grade tumors. 

## Results

A total of1736 patients were diagnosed with glial brain tumor at the department of pathology of Shohada-e Tajrish Hospital during years 2008 to 2014, of which 182 (10.5%) were found to be oligodendrogliomas. A male preponderance was observed in the sample population with 115 males (63.2%) and 67 females (36.8%), and a male to female ratio of 1.71. The mean age of the subjects was 38.5±13.36 years with a minimum of 6 and a maximum of 73 years old. The average age of male patients was slightly higher than females, yet the difference was not statistically significant (38.94±12.87 versus 37.75±14.22, p=0.563) (Table 1).

The most commonly affected site was the frontal lobe reported in 53 patients (29.1%) and the least commonly affected site was the brainstem, reported in 4 subjects (2.2%), including two tumors in pons, one in the midbrain and one involving the entire brainstem. Other affected sites based on their prevalence included the parietal lobe in 31 patients (17.0%), temporal lobe in 22 (12.1%), front oparietal area in 15 (8.2%), parieto-occipital area in 11 (6.0%), temporoparietal and frontotemporal areas each in 9 subjects (4.9%) and occipital lobe in 5 patients (2.7%). Sporadic involvement of the fourth ventricle, insula, supra sellar area, diencephalon, corpus callosum, and hypothalamus were observed in a total of 11 subjects (6.0%). The site of brain lesion was not recorded in 12 patients (6.6%). 

Considering the grade of the tumor, 108 cases (59.3%) had grade2 oligodendrogliomas and the remaining74 patients (40.7%) had grade3 anaplastic oligodendrogliomas (Table 1). The average age of patients with grade2 tumors was lower than patients with grade3 oligodendrogliomas, yet the difference was not statistically significant (37.35±11.7 versus 40.18±15.35 years, p=0.162). Furthermore, 43.5% of male subjects had grade3 anaplastic tumors while this figure was 35.8% among female subjects; however, the difference was not statistically significant (p=0.350). 

The differences in age of patients were evaluated based on the location of their tumor using the ANOVA test and Tukey’s HSD post hoc test. The results of this analysis are presented in Table 2. 

**Table 1:** Demographic Characteristics of the Patients

Variable		Count/Mean
Age (mean ± standard deviation)		38.5±13.36 years
Gender	Male	115 (63.2%)
	Female	67 (36.8%)
Location	Frontal lobe	53 (29.1%)
	Temporal lobe	22 (12.1%)
	Parietal lobe	31 (17.0%)
	Occipital lobe	5 (2.7%)
	Frontotemporal Area	9 (4.9%)
	Frontoparietal Area	15 (8.2%)
	Temporoparietal Area	9 (4.9%)
	Parieto-occipital Area	11 (6.0%)
	Sporadic involvement	11 (6.0%)
	Site not specified	12 (6.6%)
	Brainstem	4 (2.2%)
Grade	Grade 2 oligodendroglioma	108 (59.3%)
	Grade 3 anaplastic oligodendroglioma	74 (40.7%)

The results of this analysis are presented in Table 2. As indicated, the mean age of the subjects with brainstem oligodendrogliomas was significantly lower than the mean age of patients with tumors in other locations (p=0.025)

Table 3 shows the relationship between the location of the tumor, and gender of the patients and the grade of the lesion. The P value calculated using the Chi-square test for the correlation between location and grade of the tumor was 0.013 and the differences were found to be statistically significant, while the P value for the gender of the subjects was 0.427. 

**Table 2:** age of Patients Based on the Location of their Tumor (Tukey’s HSD analysis)

Location	N	Subset for alpha = 0.05	
		1	2
Brainstem	4	12.50	
Frontoparietal Area	15		34.00
Sporadic involvement	11		35.73
Site not specified	12		37.67
Parieto-occipital Area	11		37.82
Occipital lobe	5		38.40
Parietal lobe	31		39.06
Frontotemporal Area	9		40.00
Frontal lobe	53		40.58
Temporal lobe	22		40.59
Temporoparietal Area	9		42.11
Sig.		1.000	.947

Considering the grade of tumors according to their location, 11% of grade3 oligodendrogliomas were found in the frontotemporal lesions, with this being the lowest percentage, while 19.4% were found in parietal lobe lesions. The highest percentage was observed in occipital lobe lesions (80.0%) followed by the parieto-occipital lesions with a percentage of 72.7%.

However, when the other two variables were controlled through binary logistic regression analysis, none of the variables of age (p=0.104), gender (p=0.409) or location of the tumor (p=0.141) was independently predictive of a higher-grade lesion. 

**Table 3:** The Relationship between Grade of the Tumors and Their Location

		Grade		Gender		Total
		Grade 2	Grade3	Male	Female	
Location	Frontal lobe	30 (56.6%)	23 (43.4%)	32 (60.4%)	21 (39.6%)	53
	Temporal lobe	14 (63.6%)	8 (36.4%)	12 (54.5%)	10 (45.5%)	22
	Parietal lobe	25 (80.6%)	6 (19.4%)	17 (54.8%)	14 (45.2%)	31
	Occipital lobe	1 (20.0%)	4 (80.0%)	5 (100.0%)	0 (0.0%)	5
	Frontotemporal Area	8 (88.9%)	1 (11.1%)	5 (55.6%)	4 (44.4%)	9
	Frontoparietal Area	7 (46.7%)	8 (53.3%)	11 (73.7%)	4 (26.7%)	15
	Temporoparietal Area	5 (55.6%)	4 (44.4%)	6 (66.7%)	3 (33.3%)	9
	Parieto-occipital Area	3 (27.3%)	8 (72.7%)	10 (90.9%)	1 (9.1%)	11
	Sporadic involvement	5 (45.5%)	6 (54.5%)	8 (72.7%)	3 (27.3%)	11
	Site not specified	9 (75.0%)	3 (25.0%)	7 (58.3%)	5 (41.7%)	12
	Brainstem	1 (25.0%)	3 (75.0%)	2 (50.0%)	2 (50.0%)	4
Total		108 (59.3%)	74 (40.7%)	115 (63.2%)	67 (36.8%)	182
P value		0.013		0.427			

## Discussion

In this descriptive retrospective study, 181 patients with definite diagnosis of oligodendroglioma were included from patients treated at the referral center of Shohada-e Tajrish Hospital during years 2008 to 2014.

A male preponderance was observed in the sample population with a male to female ratio of 1.71, which was compatible with the results of current literature. Previous studies had also reported a higher risk of developing oligodendrogliomas among males compared to females (4, 6, 12-14, 16, 18). 

The average age of male patients was slightly higher than females, yet the difference was not statistically significant (38.94±12.87 vs. 

37.75±14.22, p=0.563). This finding was incongruent with the results reported by Fleury et al. (6) and Nielsen et al.(24). These researchers found that the peak incidence occurred at a slightly higher age in females compared to males. They speculated that this difference might have been due to the effects of female sex hormones and older age, which may have offered protective effects against developing oligodendrogliomas. 

However, their hypothesis was not confirmed and was further opposed by the current results. 

The most commonly affected site was the frontal lobe (29.1%) and the least commonly affected site was the brainstem (2.2%). Other tumor sites in order of frequency were as follows, parietal lobe (17.0%), temporal lobe (12.1%), frontoparietal area (8.2%), parieto-occipital area (6.0%), temporoparietal and frontotemporal areas (4.9%), and occipital lobe (2.7%). Therefore, the total involvement of the frontal, temporal and parietal lobes were 42.2%, 17% and 36.1%, respectively. In the study conducted by Mørk et al. in 1985, 208 patients with oligodendrogliomas were identified, among which the most frequent site of tumor was the frontal lobe consisting of53% of the cases. Parietal lobe was the second most common site reported in one-third of cases followed by the temporal lobe in one-quarter and the occipital lobe in one-sixteenth of cases (25). The commonality of tumor sites was similar between the two studies and the differences observed in the percentages was due to the fact that Mork et al. did not include subgroups of common areas between the main lobes, such as frontoparietal, parieto-occipital, temporoparietal and frontotemporal. Chin et al. also reported that the frontal lobe was the most commonly affected site involving approximately half of cases evaluated in their study, followed by the temporal lobe (26). In another survey, Shaw et al. reported the overall involvement of frontal, temporal and parietal lobes to be 50%, 42% and 32%, 

respectively(22). All these studies were congruently indicating that the most common site affected by oligodendrogliomas is the frontal lobe. 

Considering the grade of the tumor, 59.3% of patients had grade2 oligodendrogliomas and the remaining 40.7%were reported to have grade3 anaplastic oligodendrogliomas. The average age of patients with grade2 tumors was lower than patients with grade3 oligodendrogliomas, yet the difference was not statistically significant (37.35±11.7 versus 40.18±15.35 years, p=0.162).Shaw et al. reported similar frequencies for tumor grades in their research on patients referred to the Mayo clinic (22). Although the frequency of grade 3 tumors in their study was slightly higher than grade 2 oligodendrogliomas, yet they were very similar and their ratio was approximately 1. 

Another finding of the present study was that the mean age of subjects with brainstem oligodendrogliomas was significantly lower than the mean age of patients with tumors at other locations (p=0.025). A bimodal age distribution has been previously reported for the prevalence of brainstem gliomas in children and adults. Accordingly, these results can be explained by the fact that these tumors represent up to 20% of brain tumors in the pediatric population, but only 1% to 2% of adult brain tumors (27). 

There was a statistically significant correlation between location and grade of the tumor (p=0.013) and the percentage of grade3 oligodendrogliomas was lowest in frontotemporal lesions with 11.1%while the highest percentage was observed in occipital lobe lesions with 80%. However, when the 2 variables were controlled, none of the variables of age (p=0.104), gender (p=0.409) or location of the tumor (p=0.141) independently predicted a higher grade lesion.

This study was one of the few studies conducted on basic characteristics of oligodendrogliomas in Iran, evaluating a wide temporal range of 7 years. However, important variables such as the hemisphere, size of the lesions, imaging characteristics of the tumors, the clinical presentation of the patients, their treatment, and survival were not evaluated in the present study due to the lack of resources. Therefore, it is suggested that further studies should be conducted to include greater sample populations and to evaluate more of these important variables. 

## Conclusion

The incidence of oligodendrogliomas shows a male preponderance with a male/female ratio of 1.71. The most commonly affected site is the frontal lobe, followed by the parietal lobe and the temporal lobe. The mean age of subjects with brainstem oligodendrogliomas was significantly lower than mean age of patients with tumors in other locations. Age, gender or location of the tumor did not independently predict a higher grade lesion.
